# Illnesses and Deaths Among Persons Attending an Electronic Dance-Music Festival — New York City, 2013

**Published:** 2014-12-19

**Authors:** Alison Ridpath, Cynthia R. Driver, Michelle L. Nolan, Adam Karpati, Daniel Kass, Denise Paone, Andrea Jakubowski, Robert S. Hoffman, Lewis S. Nelson, Hillary V. Kunins

**Affiliations:** 1 New York City Department of Health and Mental Hygiene; 2 Epidemic Intelligence Service, CDC

Outdoor electronic dance-music festivals (EDMFs) are typically summer events where attendees can dance for hours in hot temperatures. EDMFs have received increased media attention because of their growing popularity and reports of illness among attendees associated with recreational drug use. MDMA (3,4-methylenedioxymethamphetamine) is one of the drugs often used at EDMFs (1). MDMA causes euphoria and mental stimulation but also can cause serious adverse effects, including hyperthermia, seizures, hyponatremia, rhabdomyolysis, and multiorgan failure (2,3). In this report, MDMA and other synthetic drugs commonly used at dance festivals are referred to as “synthetic club drugs.” On September 1, 2013, the New York City (NYC) Department of Health and Mental Hygiene (DOHMH) received reports of two deaths of attendees at an EDMF (festival A) held August 31–September 1 in NYC. DOHMH conducted an investigation to identify and characterize adverse events resulting in emergency department (ED) visits among festival A attendees and to determine what drugs were associated with these adverse events. The investigation identified 22 cases of adverse events; nine cases were severe, including two deaths. Twenty-one (95%) of the 22 patients had used drugs or alcohol. Of 17 patients with toxicology testing, MDMA and other compounds were identified, most frequently methylone, in 11 patients. Public health messages and strategies regarding adverse health events might reduce illnesses and deaths at EDMFs.

Festival A was planned to be held outdoors from 11 a.m. to 11 p.m. over the 3-day Labor Day weekend, with approximately 40,000 attendees each day. Admission was restricted to persons aged ≥18 years. The daily outdoor heat index was 85°F–90°F (29°C–32°C). Alcoholic beverages were sold by concessionaires to persons aged ≥21 years. Ill patrons could seek care onsite at medical tents, from which ambulances transported attendees to local EDs if necessary. As a result of the two deaths, the third day of the festival was canceled by event promoters in consultation with NYC officials.

An adverse event was defined as an ED visit among any festival A attendee ≤12 hours after the event; a severe case was defined as one with seizure, intubation, intensive care unit (ICU) admission, or death. Cases were identified by review of festival A’s list of ED transports, ED registration logs for patient aged 16–30 years at nine NYC hospitals with selected key words (i.e., intoxicated, unresponsive, seizure, altered mental status, cardiac or respiratory arrest, or concert or festival attendee), NYC Poison Control Center reports of intoxications, the NYC Office of the Chief Medical Examiner list of deaths, and DOHMH’s ED syndromic surveillance system. ED records, hospital charts, medical examiner records, and laboratory results of patients with adverse events were reviewed. Available blood and urine samples from patients were sent to an external laboratory for additional toxicology testing, including testing for synthetic club drugs. Alcohol use was defined as a positive hospital laboratory result, and drug use was defined as a positive hospital or external toxicology result. Among cases without toxicology testing, patients were considered to have used drugs or alcohol if such use was noted in the medical record. Positive toxicology from drugs administered therapeutically was excluded from analysis.

Twenty-two cases were identified, 17 from the festival A ED transport list, three from the NYC Poison Control Center database, and two from NYC ED registration logs. Median age of the 22 patients was 21 years (range = 16–29 years). Fifteen (68%) were residents of New York state, and four were residents of NYC. Four (18%) had body temperature greater than 102°F (38.9°C) (Table 1).

Among the 22 patients, 21 (95%) had used drugs or alcohol. Eleven (50%) had used alcohol with or without other drugs, and 12 (55%) had used synthetic club drugs with or without other drugs or alcohol. Among the nine severe cases, six had used synthetic club drugs only and none had used alcohol only. Biologic specimens were available for additional toxicology testing from 17 patients. MDMA was identified in one decedent, and MDMA plus methylone (a synthetic cathinone) in the other decedent. Four of 17 tested positive for methylone alone; three for methylone and MDMA; one for methylone and methamphetamine; one for methylone, methamphetamine, and cocaine; and two for MDMA alone (Table 2).

In comparison with other EDMFs occurring in NYC during September 2012–September 2014 or a 2010 New Year’s Eve EDMF in Los Angeles (4), the rates among attendees of hospital admissions and ICU admission or death per 10,000 person days did not differ significantly (Table 3). The death rate associated with festival A in 2013 also was compared with the number of unintentional poisoning deaths from all psychoactive substances in a comparable NYC age group during 2012, the most recent year that collated data were available (5). Among persons aged 15–34 years, the death rate from all psychoactive substances in NYC was 0.02/100,000 person-days, compared with 2.5/100,000 person-days at festival A in 2013.

## Discussion

This investigation identified 22 attendees with adverse events, including two deaths, associated with an EDMF; 95% of the attendees had used drugs or alcohol, and toxicology testing identified MDMA and other compounds, most frequently methylone. Drugs believed to contain MDMA are sold under the street names “ecstasy” and “molly.” These illicit substances might contain additional or substituted compounds. According to the Drug Abuse Warning Network, the number of ED visits nationally involving MDMA increased 120% during 2004–2011 (6). Although fatal drug overdoses have been reported at EDMFs, no reports regarding the rate of MDMA use at EDMFs are available, although one study reported a 5.4% prevalence of “amphetamines/MDMA” in drug assays among patrons exiting San Francisco clubs with electronic dance music events (7).

Limited information exists regarding rates of hospital admissions and deaths at EDMFs to compare with rates from the 2013 festival A. One published investigation was conducted in Los Angeles after the death of an attendee at a New Year’s Eve EDMF (4). In NYC, adverse health events at music festivals have not been routinely reported to DOHMH. However, during the summer of 2012, two ED physicians reported to DOHMH that multiple persons requiring ICU admission had been transported from an EDMF. As a result, DOHMH initiated surveillance for adverse events at EDMFs, which detected the two deaths at festival A in 2013. EDMF organizers were asked to report to DOHMH every 4 hours the number of medical tent visits and attendees transported to EDs. Hospitals were alerted in advance and reminded to report drug poisoning to the NYC Poison Control Center, and DOHMH syndromic surveillance of EDs was modified to identify visits relating to drug use and overdose.

The death rate associated with festival A in 2013 was found to be much higher than that for unintentional poisoning deaths from all psychoactive substances in a comparable NYC age group during 2012. However, without toxicology comparisons, it cannot be determined whether methylone, a compound chemically similar to MDMA with both stimulant and hallucinogenic properties and similar adverse effects, might have been the cause of the higher than expected mortality (8).

DOHMH has developed recommendations to mitigate the risk for adverse events at future EDMFs, including restricting admission to persons aged ≥18 years, employing strategies to reduce excess alcohol consumption, prohibiting the sale of mixed energy-alcohol drinks, providing readily accessible no-cost drinking water, identifying impaired patrons and bringing them to medical attention (e.g., by using roaming teams and visual inspections of attendees at entrances and exits), developing a plan to prevent heat-related illness for summer events, distributing harm-reduction messages in advance of and during events; and implementing a surveillance system to rapidly identify adverse health events including reporting ED transports to DOHMH every 4 hours.

Festival A was held again in 2014 in NYC over the Labor Day weekend. The outdoor heat index was 80°F–90°F, and there were ≤ 25,000 attendees each day. At this year’s festival A, promoters with DOHMH consultation instituted and strengthened a number of safety measures, including roaming teams of peer volunteers (one per 500 attendees), stricter entrance procedures (denying admission to ticket holders visibly under the influence of drugs or alcohol), procedures to reduce heat exposure (reduced festival hours), and required viewing of harm reduction messages before entering the festival. The DOHMH surveillance system identified 10 cases from festival A this year, including two severe cases and one death. The death was attributed to use of methamphetamine. The death occurred several hours after the event had closed for the day; future mitigation strategies might include enhanced supervision of patrons leaving the venue.

The findings in this report are subject to at least three limitations. First, data regarding adverse events or drug use for attendees not requiring ED transport were unavailable. It is known that a substantial number of persons were treated on-site and that certain persons would likely have been transported to EDs had medical treatment tents not been available. Second, information regarding additional risk factors (e.g., physical exertion, amount and frequency of drug and alcohol use, and intake of caffeine, water, and food) was limited. Third, biologic specimens were not available from all patients for external testing; for these untested patients, drug use was defined on the basis of a medical record report, which might have resulted in misclassification of the exposure. Of six patients not tested for alcohol, two reported alcohol use in the medical record. Of five patients without external toxicology testing, one reported MDMA use in the medical record.

What is already known on this topic?

MDMA (3,4-methylenedioxymethamphetamine), also known as ecstasy or molly, is an amphetamine derivative that has both stimulant and hallucinogenic effects. Although MDMA is an illicit substance, it is used recreationally, including at electronic dance-music festivals, and can cause adverse health events.

What is added by this report?

The New York City Department of Health and Mental Hygiene investigated adverse events resulting in emergency department visits among persons who attended an electronic dance-music festival held August 31–September 1, 2013 in the city. The investigation identified 22 cases of adverse events; nine were severe, including two deaths. Twenty-one of 22 patients had used drugs or alcohol. Of 17 patients with toxicology testing, MDMA and other compounds were identified, most frequently methylone, in 11 patients.

What are the implications for public health practice?

As a result of this investigation, the New York City Department of Health and Mental Hygiene and festival promoters developed multiple interventions including implementing a surveillance system for adverse events and safety measures (e.g. roaming teams of peer volunteers, stricter entrance procedures, procedures to reduce heat exposure, and required viewing of harm reduction messages before entering the festival). These interventions might help prevent adverse health events at future electronic dance-music festivals in New York City and elsewhere.

Depending on applicable state and local laws, health departments might have a role in issuing permits, determining medical service requirements, recognizing adverse health events, and guiding harm reduction messaging at EDMFs. Further study is needed of risk factors that might modify rates of adverse health events from EDMFs. In addition, study of other mass-gathering events could provide data for comparison with EDMFs.

## Figures and Tables

**TABLE 1:** Number (N = 22) and percentage of attendees transported to emergency departments after an electronic dance music festival, by selected characteristics — New York City, 2013

Characteristic	No.	(%)
**Sex**		
Female	13	(59)
Male	9	(41)
**Median age (range) (yrs)**	21 (16–29)	
**Age group (yrs)**		
<18*	2	(9)
18–20	8	(36)
≥21	12	(55)
**Residence**		
New York state	15	(68)
New York City	4	(18)
**Signs and symptoms (no. of persons tested)**		
Temperature >102°F (38.9°C)	4	(18)
Tachycardia (heart rate >100 beats/min)	14	(64)
Low sodium (sodium <135 mEq/L)	5 (18)	(23)
Acute kidney injury (creatinine >1.3 mg/dL)	4 (17)	(24)
Muscle breakdown (creatinine kinase >1,000 IU/L)	7 (7)	(100)
**Disposition**		
Treated and released at the hospital	13	(59)
Admitted to the hospital	5	(23)
Died	2	(9)
Other†	2	(9)
Severe case	9	(41)
Seizure	6	(27)
Intubated	5	(23)
Admitted to intensive care unit	5	(23)
Died	2	(9)

* The festival was restricted to those aged ≥18 years, however two persons were reported as aged <18 years in the medical records.

† One person left before being evaluated by a physician, and one person left against medical advice.

**TABLE 2:** Number (N = 22) and percentage of attendees transported to emergency departments after an electronic dance music festival, by drug and alcohol use — New York City, 2013

Drug and alcohol use	No.	(%)	Severe cases (n = 9)		Nonsevere cases (n = 13)	
	
			No.	(%)	No.	(%)
Any drug or alcohol use	21	(95)	9	(100)	12	(92)
Alcohol use with or without other drugs	11	(50)	3	(33)	8	(62)
Alcohol use only	6	(27)	0	(0)	6	(46)
Synthetic club drug use with or without other drugs or alcohol	12	(55)	8	(89)	4	(31)
Synthetic club drug use only	9	(41)	6	(67)	3	(23)
Marijuana use with or without other drugs or alcohol	3	(14)	1	(11)	2	(15)
Cocaine use with or without other drugs or alcohol	1	(5)	1	(11)	0	(0)

**TABLE 3:** Numbers and rates of hospitalization and intensive care unit (ICU) admission or death among attendees of selected electronic dance music festivals — New York City and Los Angeles, 2010–2014

Electronic dance music festival (year)	Person-days attendance	Transported to ED*	Treated in medical tent*	Total hospitalizations†		ICU admission or death†	
				
	No.	No.	No.	No.	Rate per 10,000 person-days	No.	Rate per 10,000 person-days
Los Angeles New Year’s Eve festival (2010)	45,000	18	NA	3	0.67	1§	0.22
New York City festival A (2012)	106,000	135	1,100	11	1.04	7	0.66
New York City festival A (2013)	80,000	18	964	5	0.63	7	0.88
New York City festival B (2013)	90,000	39	252	4	0.44	4	0.44
New York City festival A (2014)	58,000	10	NA	1	0.17	2	0.34

**Abbreviations:** ED = emergency department; NA = not available.

* Number of persons transported to ED and treated in medical tent as reported by medical providers.

† p-values were >0.05 for comparison of rates among all festivals.

§ Los Angeles reported the death of one attendee; however, this death did not meet the case definition because death occurred >12 hours after the festival ended.
